# Inflammatory Fibroid Polyp or Vanek Tumor as an Uncommon Cause of Adult Intussusception: Diagnostic and Surgical Considerations

**DOI:** 10.7759/cureus.85619

**Published:** 2025-06-09

**Authors:** Anaida Xacur Trabulce, Begoña Llaca Morfin, Gessner Casas Fuentes, Atl Simon Arias Rivera, Andoni Vicente Eguia

**Affiliations:** 1 General Surgery, Hospital Angeles Lomas, Huixquilucan, MEX

**Keywords:** bowel obstruction, inflammatory fibroid polyp, small bowel intussusception, submucosal tumor, vanek's tumor

## Abstract

Inflammatory fibroid polyps (IFPs) or Vanek tumors are rare benign neoplasms of the gastrointestinal tract, with an unknown pathogenesis, that, despite their harmless nature, can lead to small bowel intussusception - a rare cause of intestinal obstruction in adults. In this article, we present the case of a 69-year-old man who arrived at the emergency department with vague abdominal discomfort. Imaging revealed a jejunojejunal intussusception caused by a polypoid mass, which was successfully treated through laparoscopic-assisted bowel resection. Histopathology and immunohistochemistry confirmed the diagnosis of IFPs. A comprehensive literature review was conducted, identifying 22 previously published cases to better understand the clinical profile and management of this rare condition. It highlights the importance of raising awareness among clinicians about this unusual disease so that it can be recognized and receive prompt surgical treatment as key factors to prevent complications and ensure full recovery.

## Introduction

Inflammatory fibroid polyps (IFPs) are uncommon, benign, non-epithelial tumors that mostly present as polypoid masses. They may develop anywhere in the gastrointestinal tract, but they are commonly found in the stomach (70%), specifically in the antrum, followed by the small bowel in the ileum [1-4]. These tumors arise in the submucosa and have an idiopathic nature [1,5]. The IFP was first described in 1949 by Czech pathologist Josef Vaněk (1915-1990) as a “submucosal gastric granuloma with eosinophilic infiltration” [5-9]. The exact pathogenesis of IFPs is unknown despite their specific histopathologic characteristics, described as a proliferation of spindle-shaped cells within a fibromyxoid stroma and prominent eosinophilic inflammatory infiltrate. A distinctive feature is a "whorled" or "onion-skin" appearance of stromal cells around blood vessels [2,5,10-12].

Vanek tumors contribute to less than 1% of all tumors of the gastrointestinal tract [1-4].

Most IFPs are incidentally detected during endoscopy, surgery, or imaging, because of their silent evolution. There are cases in which these tumors are located in the small bowel, and when they exceed 2-3 cm in size, they may cause obstructive symptoms. Intussusception is a rare condition in adults defined as the invagination of a proximal segment of the intestine into a distal segment. Intussusception accounts for only 1-5% of bowel obstructions, most commonly related to tumors, with benign lesions representing approximately 80% of all cases [6,13-15].

Preoperative contrast-enhanced CT imaging is essential, even though radiologic features of Vanek tumor are nonspecific, revealing only an intraluminal mass or intussusception, sometimes described as a sausage or sandwich sign and target or “bullseye” sign. The definitive diagnosis is based on histopathologic evaluation and immunohistochemistry, and for IFPs, the immunohistochemical markers are as follows: positive for CD34, negative for CD117 (c-kit), DOG1, S100, desmin, and alpha smooth muscle actin (SMA). This immunoprofile is helpful in differentiating IFPs from other neoplasms in the gastrointestinal tract, including gastrointestinal stromal tumors (GISTs), leiomyoma, and neural tumors [9,16,17].

The treatment of choice is surgical excision in symptomatic patients or when complications such as intussusception or obstruction occur. Both open and minimally invasive surgery, even in complex cases, have been described with no recurrence or malignant transformation after the resection because of the benign nature of these neoplasms [2,18,19].

Our case illustrates the typical presentation of a small intestine intussusception causing obstructive symptoms secondary to IFPs, requiring emergency surgical intervention.

A comprehensive literature review was conducted using PubMed and ScienceDirect, identifying only 20 published articles reporting IFPs of the small intestine as a cause of intussusception in adult patients, with the earliest case published in 2011.

## Case presentation

**Figure 1:**
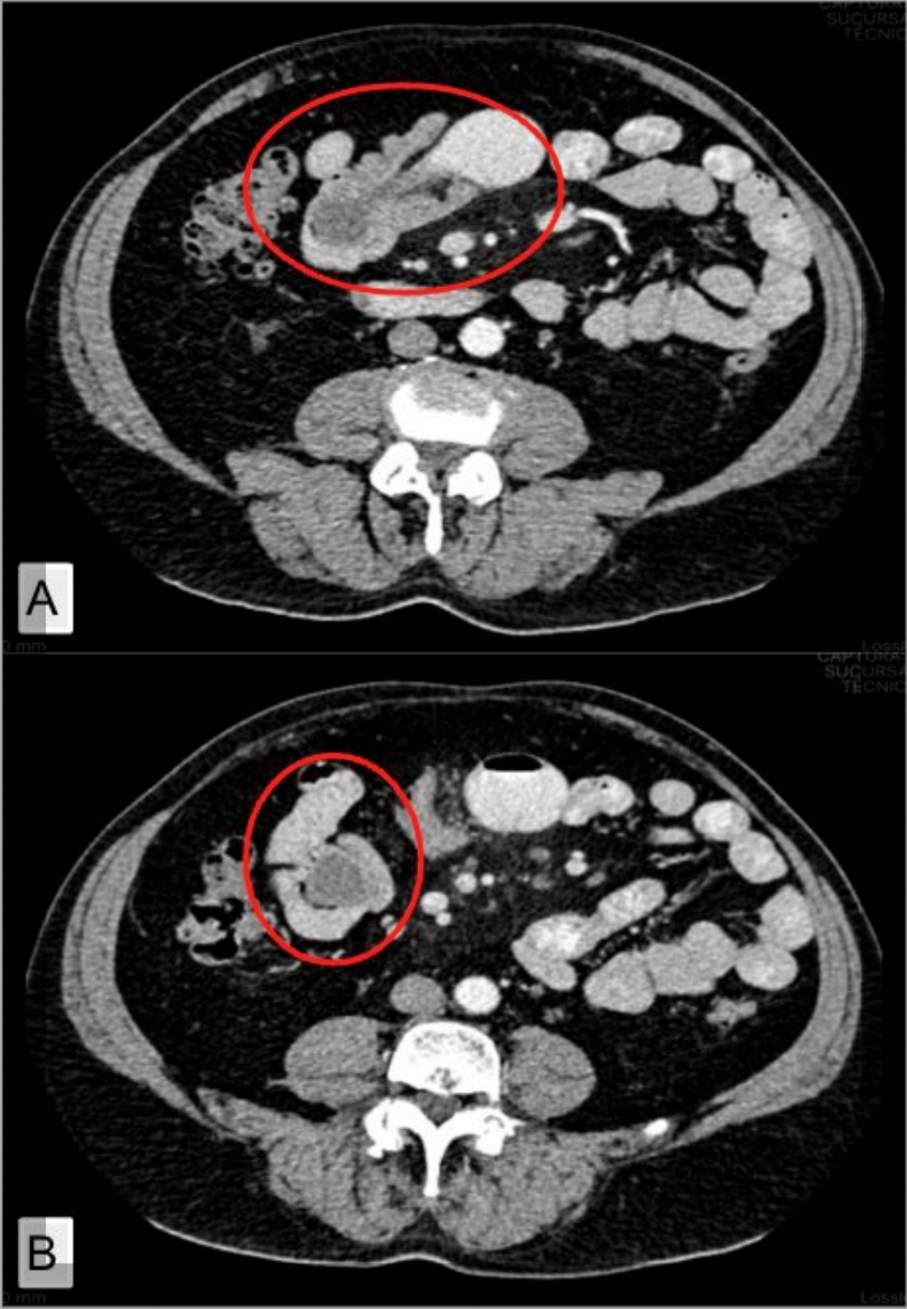
Computed tomography of the abdomen and pelvis with IV contrast A polypoid lesion of the distal jejunum that causes small bowel intussusception, considered the first possibility of a gastrointestinal stromal tumor. A) Red circle - sausage or sandwich sign. B) Red circle - target or “bullseye” sign.

A 69-year-old male was referred to the emergency department due to abdominal pain. During anamnesis, he reports acute colicky pain in the lower abdominal quadrants that migrates to the periumbilical region, intensity described with a punctuation on the pain scale of 4 out of 10. He denies any presence of nausea, vomiting, weight loss, fever, or diaphoresis. In the clinical examination, we find a globular abdomen due to panniculus, hypoactive peristalsis, tympanic percussion of the colic frame, and pain on the palpation of the mesogastrium, without signs of peritoneal irritation. A computed tomography of the abdomen and pelvis with IV contrast revealed a polypoid lesion located in the distal jejunum, which caused a small bowel intussusception, cholelithiasis, and uncomplicated diverticular disease (Figure 1).

**Figure 2:**
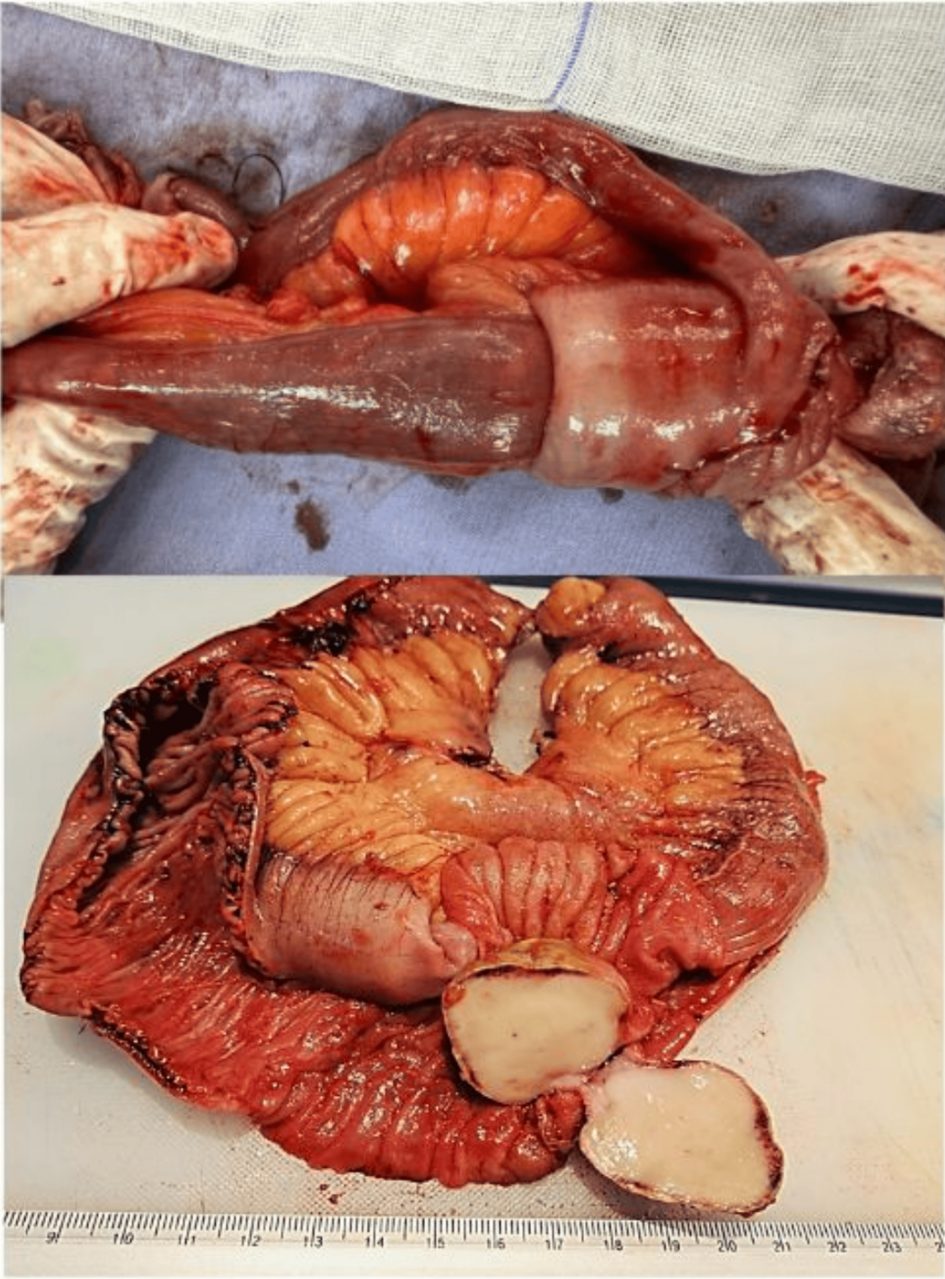
Intraoperative pathological examination findings Intestinal intussusception located 250 cm from the Treitz angle, with a 4.1 × 3.4 cm tumor exhibiting extensive ulceration on the superficial portion. Marked edema and vascular congestion are present, along with areas of recent microhemorrhage in the intestinal wall.

**Figure 3:**
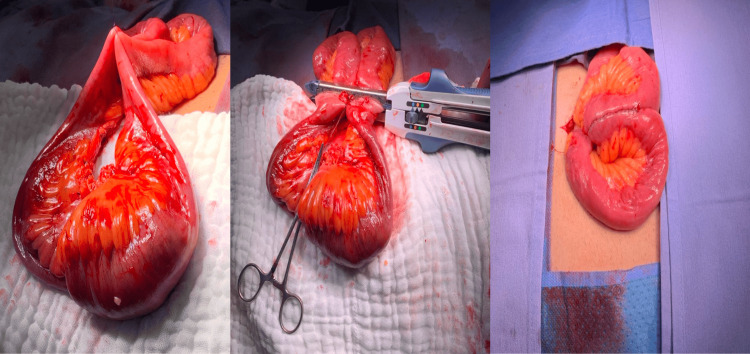
Side-to-side entero-enteric anastomosis using a linear stapler The staple line is clearly visualized, demonstrating precise alignment and a tension-free configuration between the adjacent small bowel loops.

On account of the imaging and clinical features of the patient, an exploratory laparoscopy was performed. The following intraoperative findings were identified: intestinal intussusception located 250 cm distal to the Treitz angle, and within the intestinal lumen, a tumor measuring approximately 3.4 x 4.1 cm was found, without evidence of free fluid or intestinal perforation in the abdominal cavity (Figure 2). Because of the impossibility of intracorporeal reduction, it was necessary to expand the umbilical incision by 4 more centimeters to facilitate the exteriorization of the involved loop. A 14.5-cm small bowel resection was performed. Subsequently, a side-to-side entero-enteric anastomosis using a linear stapler was executed (Figure 3). The anastomosis was reduced back to the abdominal cavity, followed by a complementary laparoscopic cholecystectomy.

**Figure 4:**
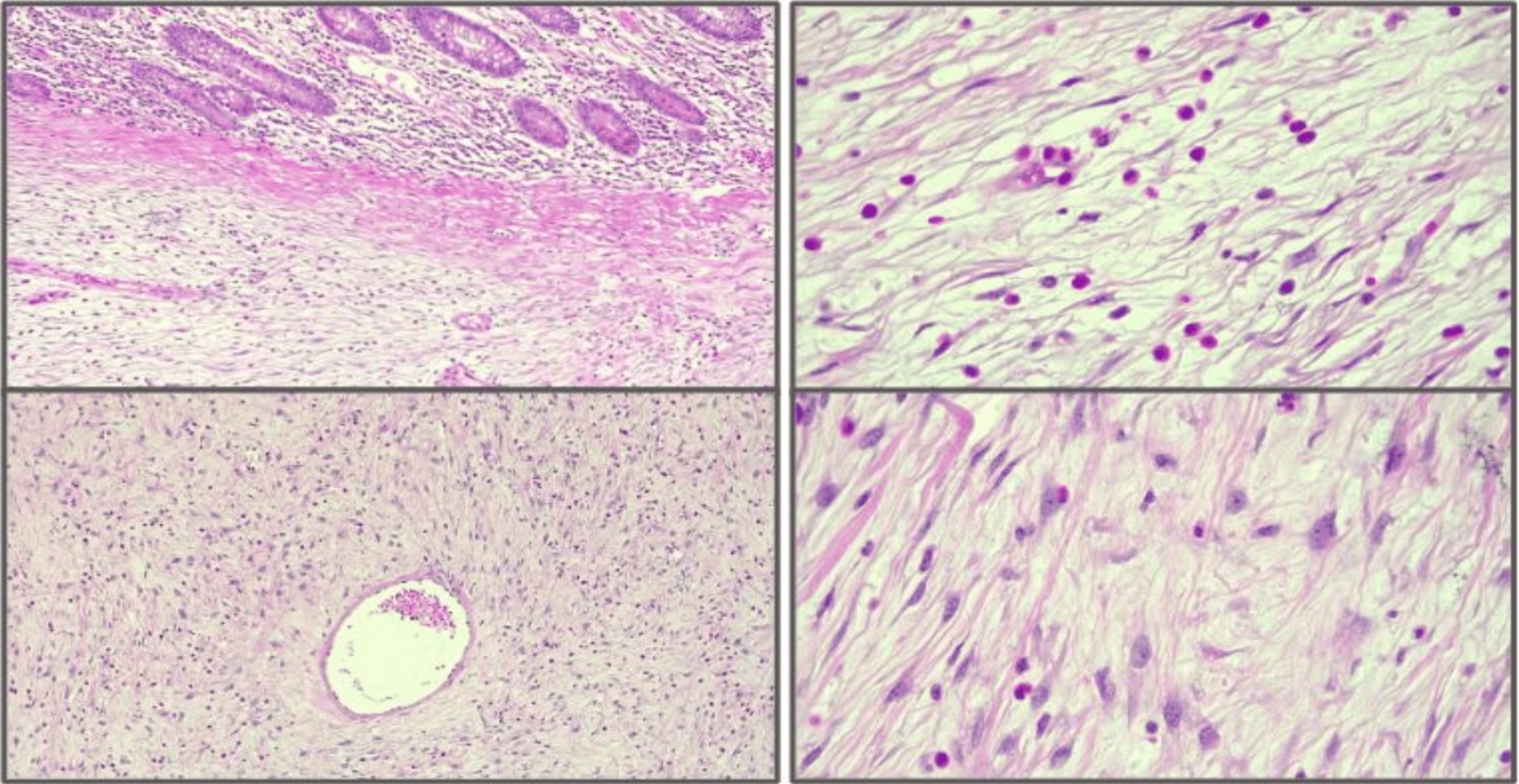
Histopathology Fibromyxoid tissue with inflammatory infiltration, spindle-shaped or stellate cells, and inflammatory cells, typically eosinophils. Thin-walled blood vessels are arranged in an "onion-skin" pattern. The lesion was diagnosed as an inflammatory fibroid polyp. A staining method was used for histopathological evaluation, although the specific technique was not disclosed.

**Figure 5:**
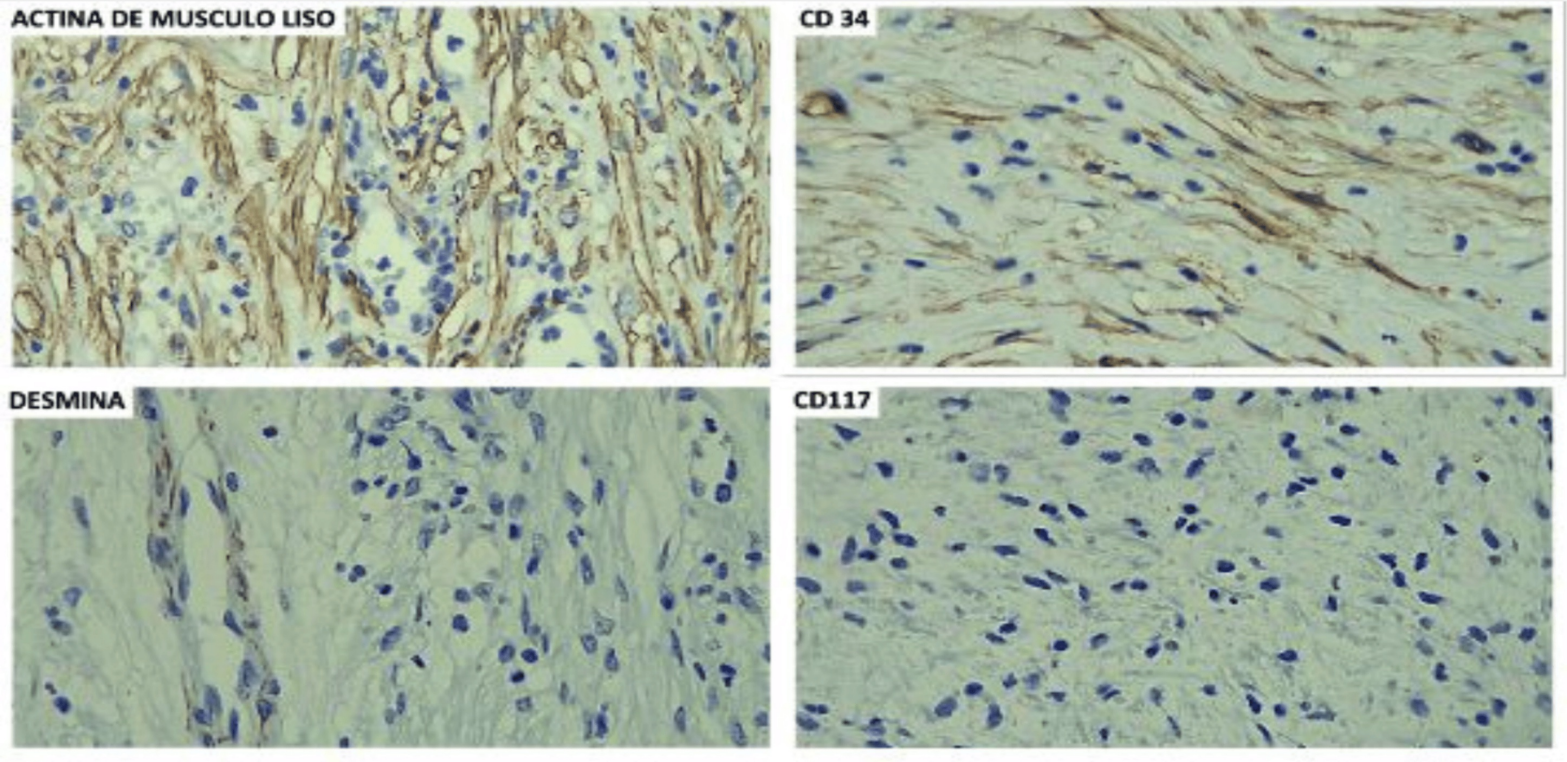
Immunohistochemistry Smooth muscle actin (SMA) positive, CD34 positive, CD117 negative, DOG-1 negative, ALK negative, desmin negative, and MDM-2 negative. Proliferation index: 1%. Ki-67 staining was performed with a result of <1%; however, it is not shown.

The intestinal resection segment and gallbladder specimens were sent to pathology, and the histopathological analysis identified a polypoid tumor with morphological and immunophenotypic findings consistent with an inflammatory fibroid polyp (Vanek tumor) characterized by proliferative stromal cells (Figure 4). The 4.1 x 3.4 cm lesion exhibited extensive superficial ulceration, concomitant with marked edema, vascular congestion, and focal areas of recent microhemorrhage within the intestinal wall. A few days after the surgery, the immunohistochemical staining revealed the tumor to be positive for smooth muscle actin and CD34, and while negative for CD117, DOG-1, ALK, desmin, and MDM-2, the Ki-67 proliferation index was 1% (Figure 5). The postoperative recovery was uneventful, resulting in discharge after 72 hours.

## Discussion

**Figure 6:**
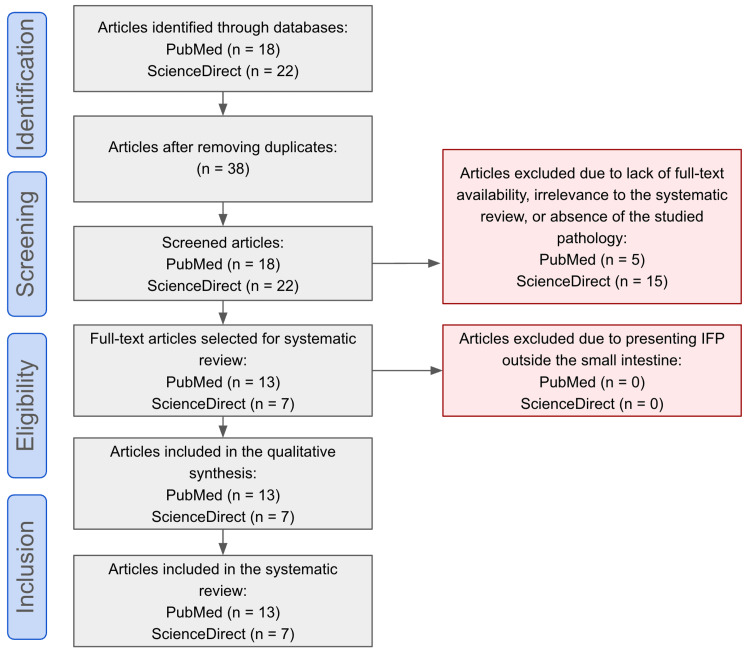
PRISMA flow diagram Article selection process using the Preferred Reporting Items for Systematic Reviews and Meta-Analyses (PRISMA)

A search of the english literature was performed by the authors (AX, BL, GC y SA) on the open-access databases PubMed and ScienceDirect, using the following algorithm ((vanek tumor) AND (inflammatory fibroid polyp)) AND (small bowel intussusception) applying more specific filters to include papers published between the years 2011-2023, incorporating review articles, research articles and case reports, in the English language. The Preferred Reporting Items for Systematic Reviews and Meta-Analyses 2020 (PRISMA) guidelines were followed, and the search included the keywords “Vanek tumor” and “inflammatory fibroid polyp”. Only cases involving small intestine localization with intestinal intussusception in adult patients were considered. The initial search included 40 articles, and 38 remained after removing duplicates. After that, 20 papers were excluded due to lack of full-text availability, irrelevance to the systematic review, or absence of the studied pathology. A total of 20 reports meeting the selection criteria were identified, covering the period from 2011 to 2023 (Figure 6). 

After full text analysis, information extraction was directed to the identification of age, sex, clinical presentation, the presence or absence of palpable mass, radiological tools, immunohistochemistry, surgical approach, location, and size of Vanek tumors (Table 1). Notably, while Figure 1 accounts for 20 studies, Table 1 comprises 22 cases. This discrepancy arises from the inclusion of two cases reported by Carvalho et al. [5] and the addition of our own case as the 22nd.

**Table 1:** Literature review General characteristics of the 22 cases of small bowel intussusception secondary to the inflammatory fibroid polyp reported between 2011 and 2023.

Reference	Age	Sex	Clinical Presentation	Palpable Mass	Radiological Tools	Immunohistochemistry	Surgical Approach	Location	Size
Chaima et al. [2]	35 years	Male	Abdominal pain for 2 weeks, intestinal obstruction for 3 days	No	Non-contrast abdominal CT: intestinal intussusception	CK7 (-), DOG1 (-)	Exploratory laparotomy + 30 cm bowel resection + side-to-side primary anastomosis	Ileum	4 cm
Park et al. [4]	23 years	Male	Diffuse abdominal pain for 3 days progressing to epigastric pressure pain, constipation, nausea, and vomiting	No	Non-contrast abdominal CT: jejunojejunal intussusception	CD34 (+), CD117 (−), BCL-2 (−), DESMIN (−), SMA (−), S-100 (−), Ki-67 1–5%	Exploratory laparoscopy + intestinal resection + side-to-side linear stapled anastomosis	Jejunum	(1) 5 x 3 x 3 cm, (2) 2.5 x 2.3 x 1.2 cm
Johan et al. [13]	41 years	Male	Diffuse abdominal pain and distension for 1 month, followed by obstipation, nausea, fecaloid vomiting, weight loss and anorexia for 3 days	No	Contrast-enhanced abdominopelvic CT: ileo-ileal intussusception	CD34 (+), CD117 (-)	Exploratory laparotomy + bowel resection + primary anastomosis + mesenteric lymph node dissection	Ileum	4 x 4 x 3.6 cm
Nonose et al. [18]	56 years	Female	Postprandial intermittent cramping abdominal pain for 45 days	No	Oral contrast-enhanced abdominopelvic CT: ileo-ileal intussusception	CD34 (+), CD117 (-), S100 (-), ACTIN (-), DESMIN (-)	Exploratory laparotomy + bowel resection + end-to-end primary anastomosis + peritoneal lavage	Ileum	4.5 cm
Al Taei et al. [3]	47 years	Female	Cramping abdominal pain, nausea, and vomiting for 4 days	No	Contrast-enhanced abdominopelvic CT: ileo-ileal intussusception	Not available	Exploratory laparotomy + 16 cm bowel resection + side-to-side anastomosis	Ileum	3.5 x 3 x 3 cm
Carvalho et al. [5]	41 years	Female	Cramping lower quadrant abdominal pain for 6 hours, nausea with one episode of vomiting and diarrhea	No	Abdominal ultrasound: positive for intestinal intussusception. Contrast-enhanced abdominopelvic CT confirmed bowel wall thickening of 35 mm	CD34 (+)	Exploratory laparoscopy + 24 cm bowel resection	Small intestine	4.8 cm
Carvalho et al. [5]	51 years	Female	Cramping right upper quadrant abdominal pain for 2 months and nausea	No	Contrast-enhanced abdominopelvic CT: ileo-ileal intussusception	CD34 (+)	Exploratory laparotomy + 22 cm bowel resection	Ileum	5.5 cm
Jan et al. [7]	60 years	Male	Generalized abdominal pain, predominantly in the left iliac fossa, moderate intensity, associated with nausea and anorexia	No	Simple abdominopelvic CT: ileo-ileal intussusception	CD34 (+)	Exploratory laparotomy + bowel resection + end-to-end primary anastomosis	Ileum	4.6 x 3.6 cm
Maya et al. [15]	82 years	Female	Generalized abdominal pain and distension for 48 hours	No	Oral contrast-enhanced abdominopelvic CT: intestinal intussusception	CD34 (+), VIM (+), S100 (-), ACTIN (-), DESMIN (-)	Exploratory laparotomy + bowel resection + end-to-end primary anastomosis	Ileum	5 cm
Neishaboori et al. [19]	40 years	Female	Progressive cramping abdominal pain, mostly postprandial, nausea and vomiting for 3 days	No	Abdominal ultrasound: positive for intestinal intussusception	CD34 (+)	Exploratory laparotomy + bowel resection + end-to-end primary anastomosis	Jejunum	18 x 5 x 1 cm
Gadoura et al. [1]	32 years	Female	Epigastric abdominal pain, nausea, vomiting, and diarrhea for 2 weeks	No	Non-contrast abdominal CT: intestinal intussusception	Not available	Exploratory laparotomy + 80 cm bowel resection + end-to-end anastomosis + lymph node dissection	Ileum	4 x 3 cm
Akbulut [11]	38 years	Female	Abdominal pain, nausea, and vomiting for 10 days	No	Abdominal ultrasound: positive for intussusception due to tumor mass	CD117 (-), SMA (-), DESMIN (-), S100 (-), CD34 (-)	Exploratory laparotomy + 20 cm bowel resection + end-to-end anastomosis	Ileum	4 x 4 cm
Ivaniš et al. [14]	38 years	Not specified	Generalized abdominal pain localized to the right hemiabdomen, worse postprandially, with diarrhea and anorexia	No	Abdominal ultrasound: positive for intussusception due to tumor mass	Not available	Exploratory laparotomy + bowel resection	Small intestine	3.5 cm
Forasté-Enríquez et al. [6]	58 years	Female	Cramping epigastric pain for 4 months associated with eating, weight loss (12 kg). Presented to ED with worsened pain, abdominal distention, diarrhea, nausea, vomiting	Yes	Contrast-enhanced abdominopelvic CT: ileo-ileal intussusception	CD34 (+), SMA (-), ALK1 (-), CD117 (-), Ki67 (-)	Exploratory laparotomy + 70 cm bowel resection + end-to-end primary anastomosis	Ileum	6.3 x 2.9 cm
Khanduri et al. [9]	41 years	Male	Sudden abdominal pain, nausea, vomiting, and obstipation for 24 hours	Yes	Contrast-enhanced abdominopelvic CT: ileo-ileal intussusception	Not available	Exploratory laparoscopy + right hemicolectomy + ileocolic anastomosis + en bloc lymph node resection (14 nodes)	Ileum	3 x 3 cm
Adams et al. [12]	61 years	Female	Diarrhea and hematochezia for 2 weeks, then right lower quadrant abdominal pain for 2 days	No	Contrast-enhanced abdominopelvic CT: ileo-ileal intussusception	CD117 (-), SMA (-), CD34 (-), DESMIN (-), S100 (-), DOG1 (-)	Exploratory laparoscopy + infraumbilical mini-laparotomy + 22 cm bowel resection + side-to-side stapled anastomosis	Ileum	7.5 x 4.5 x 3.2 cm
Jacob et al. [8]	37 years	Female	Generalized cramping abdominal pain for 4 days, vomiting and diarrhea	No	Simple abdominopelvic CT: ileo-ileal intussusception	SMA (+), CD34 (-), CD117 (-), DOG1 (-), ALK (-), DESMIN & KERATIN (-)	Diagnostic laparoscopy converted to exploratory laparotomy + 30 cm bowel resection + side-to-side ileocolic stapled anastomosis	Ileum	3 cm
Joyce et al. [11]	62 years	Male	Sudden cramping abdominal pain and abdominal distension for 12 hours	No	Simple abdominopelvic CT: jejuno-jejunal intussusception	CD117 (-), SMA (-), CD34 (-), VIM (+)	Exploratory laparotomy + 33 cm bowel resection + end-to-end anastomosis	Jejunum	4.2 x 2.5 cm
Fabbri et al. [16]	62 years	Male	Cramping abdominal pain for 40 days	No	Contrast-enhanced abdominopelvic CT: intestinal intussusception	Not available	Exploratory laparotomy + 10 cm bowel resection + side-to-side primary anastomosis	Jejunum	3.5 cm
Sakran et al. [17]	40 years	Male	Intermittent generalized abdominal pain and nausea	No	Oral contrast-enhanced abdominopelvic CT: intestinal intussusception	CD34 (+), VIM (+), S100 (-), DOG1 (-), ACTIN (-), DESMIN (-)	Exploratory laparoscopy + 10 cm bowel resection + side-to-side primary anastomosis + partial vertical gastrectomy with stapler	Ileum	Not specified
Paramythiotis et al. [20]	42 years	Female	Diffuse abdominal pain with vomiting and diarrhea for 7 days	No	Contrast-enhanced abdominopelvic CT: ileo-ileal intussusception	Not available	Exploratory laparotomy + right hemicolectomy	Ileum	Not specified
Xacur Trabulce et al. (Present study)	69 years	Male	Intermittent cramping abdominal pain starting in lower quadrants and migrating to periumbilical area for 30 days	No	Oral/IV contrast-enhanced abdominopelvic CT: distal jejunal intussusception due to polypoid lesion	ACTIN (+), CD34 (+), CD117 (-), DOG1 (-), ALK (-), DESMIN (-), MDM-2 (-), proliferation index 1%.	Exploratory laparoscopy + 15 cm bowel resection + side-to-side enteroenteric anastomosis with linear stapler + laparoscopic cholecystectomy	Jejunum-Ileum	3.4 x 4.1 cm

**Figure 7:**
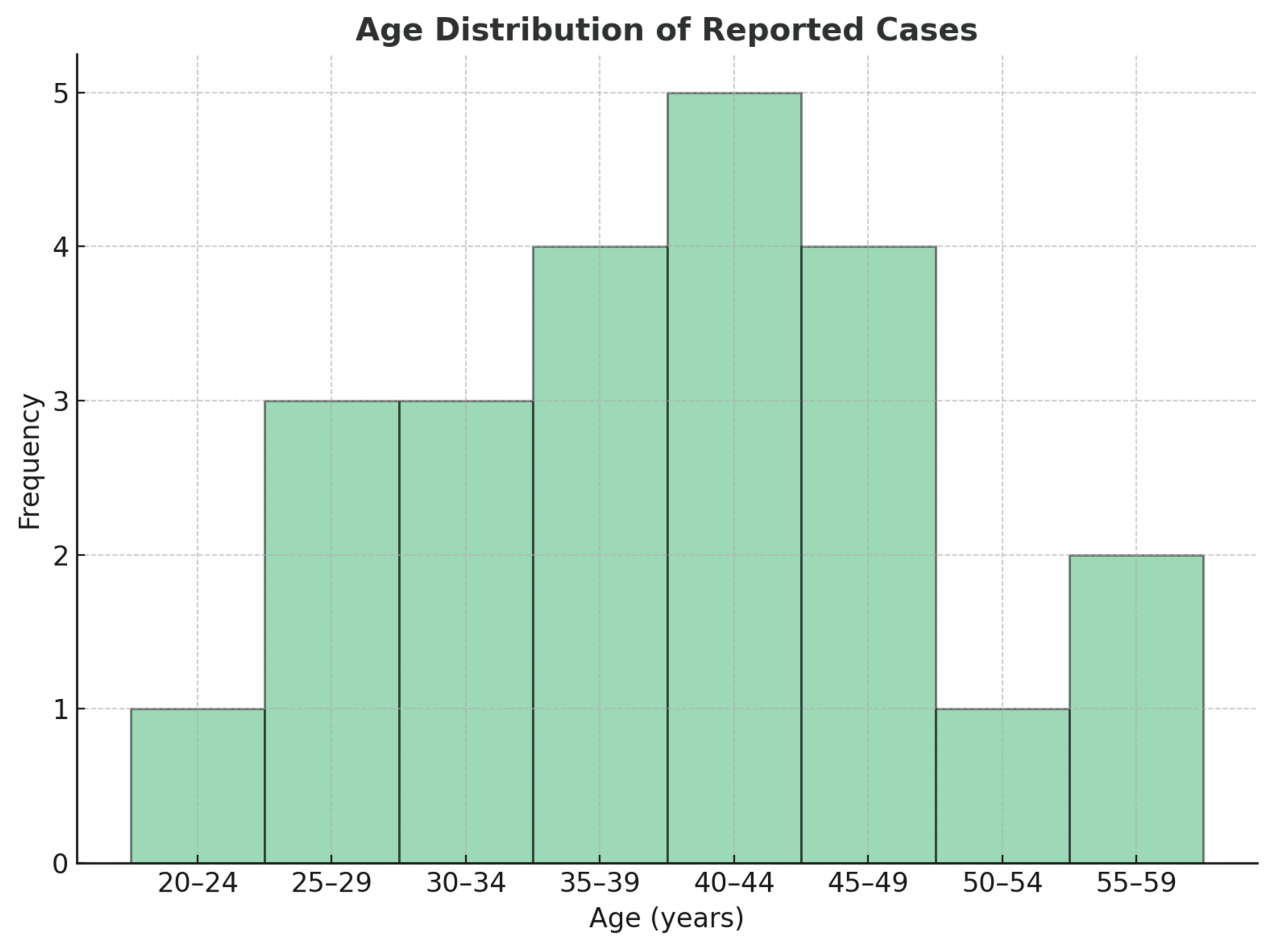
Histogram - age distribution of the reported cases Most patients were in their fourth to fifth decade of life, predominantly between 40 and 45 years. Sources: Refs [1-20]

**Figure 8:**
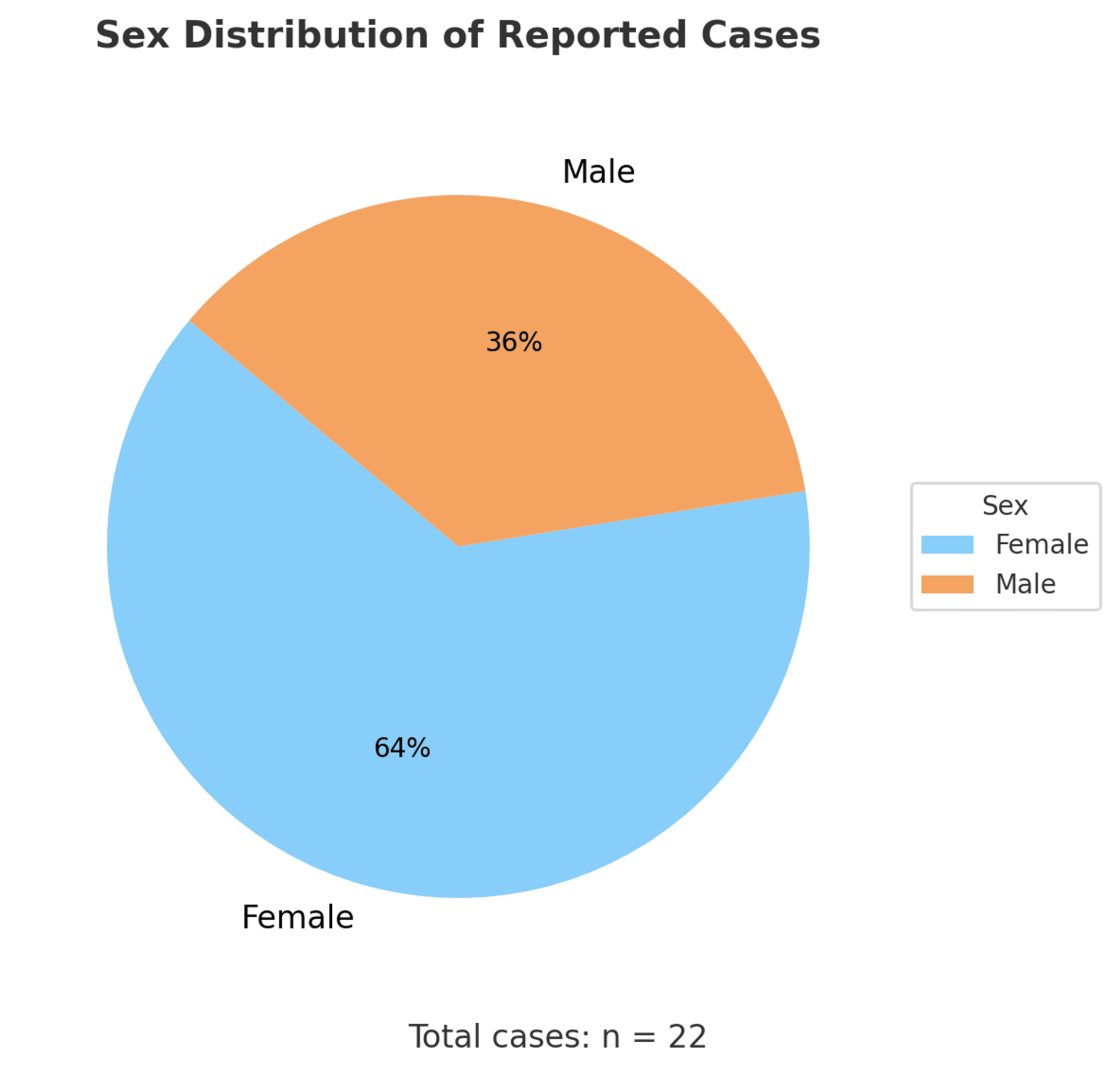
Pie chart - sex distribution of the reported cases 63.6% of the total cases occurred in females. Sources: Refs [1-20]

**Figure 9:**
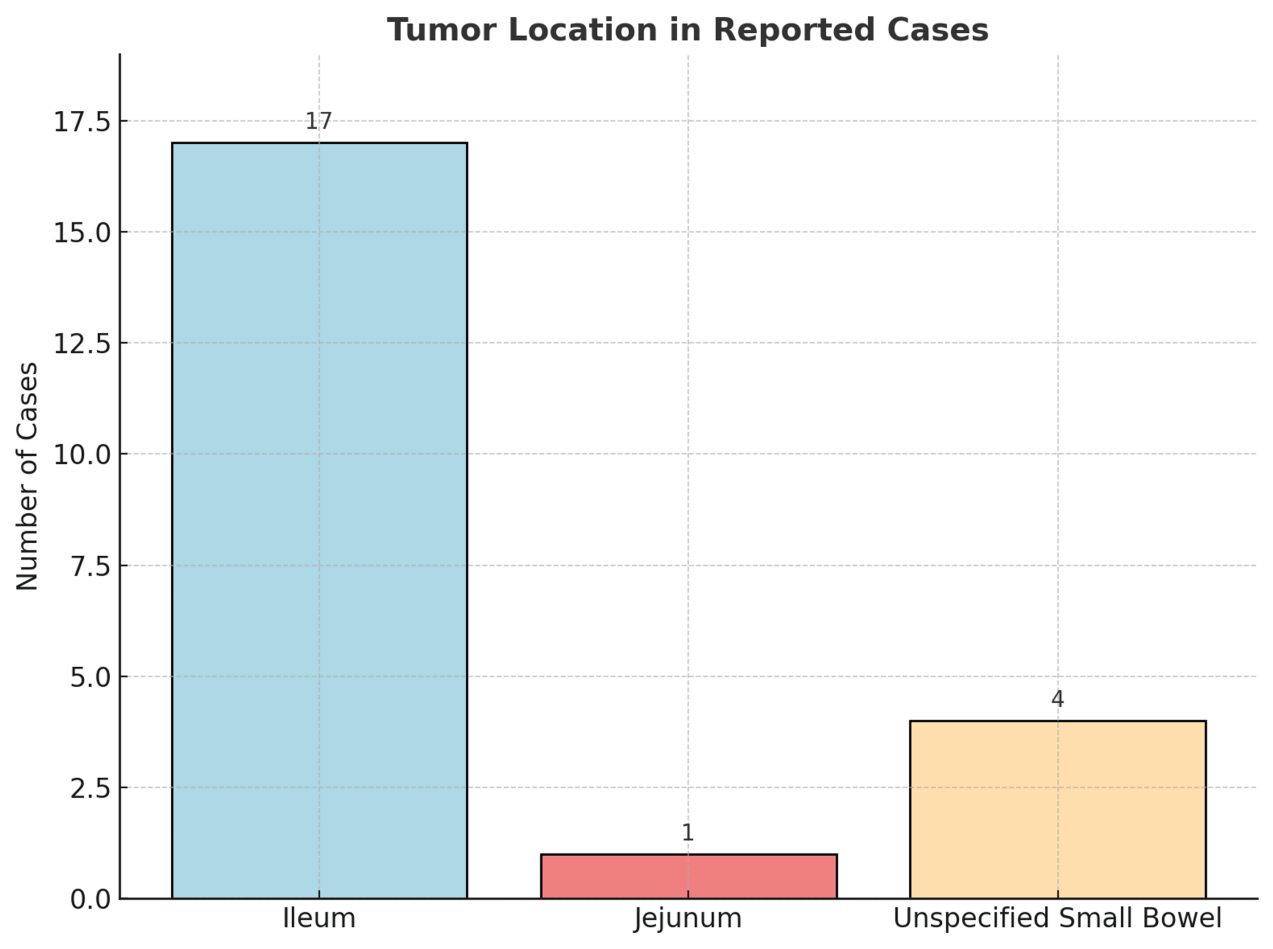
Bar chart - tumor location in the reported cases The ileum was significantly the most frequent location. Sources: Refs [10,14]

**Figure 10:**
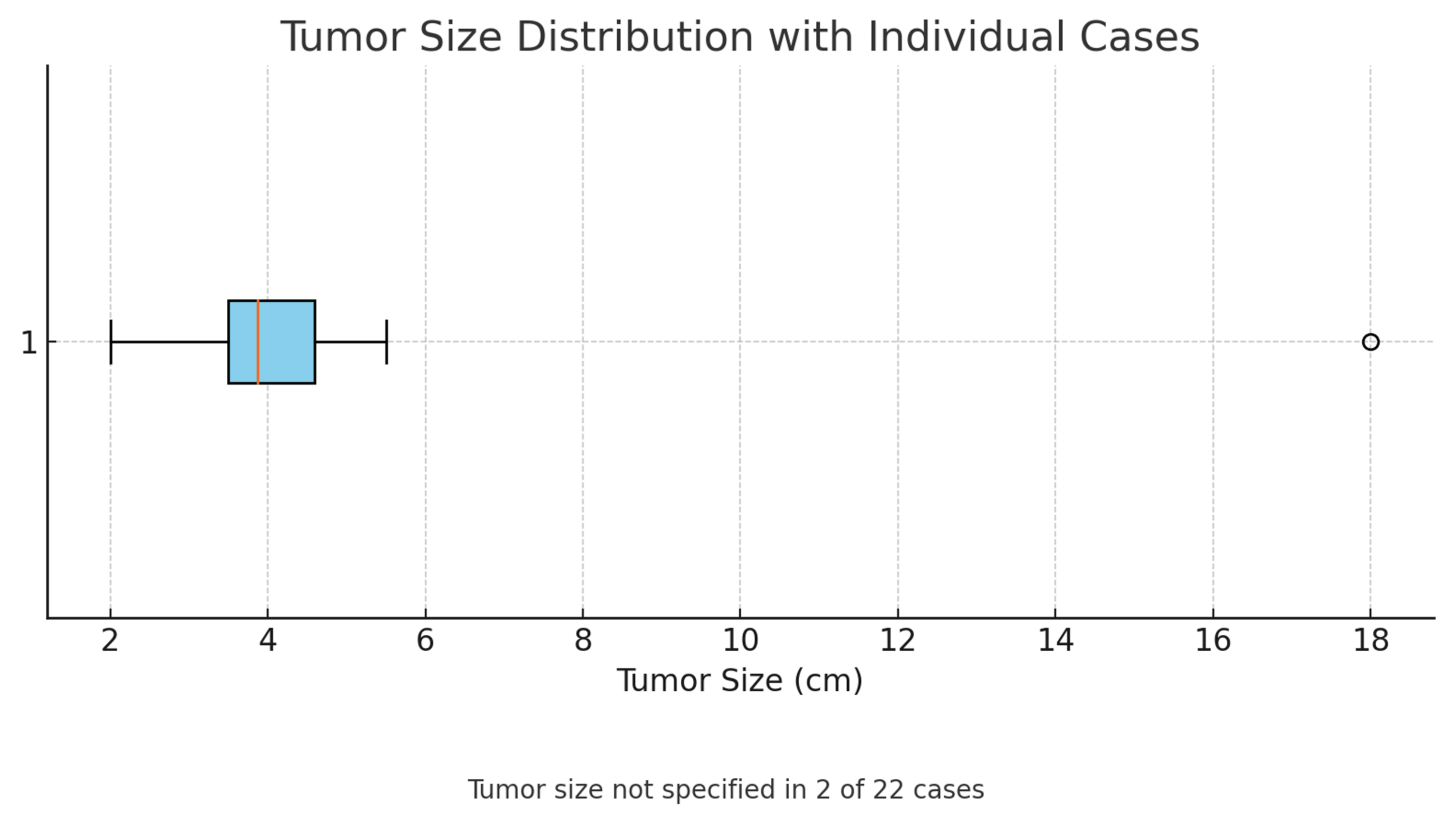
Box plot - distribution of tumor size (cm) The tumor size ranged from 1.9 to 18 cm, with most lesions measuring between 3 and 5 cm. The mean tumor diameter was approximately 4.0 cm. Tumor size was not reported in two of the 22 cases.

An analysis of 22 clinical cases was performed, and it is clear that the typical presentation of IFPs predominates in adults during the fourth to fifth decade of life (Figure 7), with a higher incidence in females (Figure 8) [1-20]. Vanek tumors are most commonly located in the stomach, followed by the small intestine, and rarely in the colon (Figure 9) [10,14]. Most times, these neoplasms are asymptomatic, and their discovery is often incidental during endoscopy or abdominal surgery. Although some patients present to the emergency department with a clinical presentation of bowel obstruction, with colicky abdominal pain representing the cardinal symptom - with variable intensity proportional to the degree of obstruction and presence of bowel ischemia [2,3,5,6]. Patients can also experience constipation, abdominal distension, nausea, vomiting, and, less frequently, diarrhea, weight loss, or gastrointestinal bleeding [1-3,5,7,8]. Tumor size presentation among the 22 cases ranged between 1.9 and 18 cm; most tumors measured between 3 and 5 cm, as illustrated in the box plot (Figure 10). Tumor size was not specified in two cases. The plot excludes one extreme outlier (18 cm) for visual clarity. This finding supports that IFPs can vary in size, although they often remain asymptomatic until they cause mechanical complications [2,6,10].

Literature implies that approximately 50% of cases may present with a palpable abdominal mass on physical examination [10]; however, only two patients in the review exhibited this clinical finding. Due to the presence of intestinal ischemia, ulceration, or perforation, additional signs will develop, such as mucocutaneous pallor, tenderness, guarding, rebound tenderness or tachycardia, progressing to hemodynamic instability, septic shock, and even death [9,12,13].

As part of the diagnostic algorithm, abdominal radiography is useful to identify the presence of bowel obstruction and free abdominal air, suggesting that perforation with the “seagull sign” can be recognized in a thorax radiography. In the abdominal ultrasound, some specific findings of intussusception might be present, just like the classic “target” or “bullseye” sign, with a reported sensitivity of 98-100% and specificity of 88-89% [10,14,20]. Notwithstanding, contrast-enhanced abdominal computed tomography remains the preferred imaging modality for adult intussusception. Typical findings in the CT scan include the “target” or “bullseye” sign, “sausage”- or “sandwich”-shaped masses, and signs of complications such as bowel obstruction, free fluid, pneumoperitoneum, intestinal pneumatosis, or mural thickening >35 mm [5,7,13]. Our case, along with those reported by Carvalho et al. [5] and Forasté-Enríquez et al. [6], provides an example of a small bowel intussusception that required surgical intervention.

Definitive diagnosis relies on histopathological examination and immunohistochemistry. Microscopic findings include fibromyxoid tissue infiltrated by eosinophils, spindle or stellate mesenchymal cells, thin-walled blood vessels in concentric (“onion-skin”) arrangements, and sometimes multinucleated giant cells [1,4,11,16]. Most IFPs express CD34 and lack markers such as CD117 (c-KIT) and S100, assisting in their differentiation from GISTs, leiomyomas, and neural tumors [8,10,14].

Complete excision by open or minimally invasive surgery remains the standard treatment for symptomatic Vanek tumors, and although when the lesions are small and accessible, endoscopic removal can be considered. Choosing the appropriate approach depends on tumor location, evidence of perforation or ischemia, and the degree of suspicion for malignancy [9,15,17]. Once the tumor is fully removed, recurrence is virtually nonexistent, and there is no need for further treatment [10,18,19].

## Conclusions

Vanek tumors located in the small intestine are uncommon, benign lesions that are generally asymptomatic but can occasionally cause intussusception, leading to bowel obstruction in adults. While they are more frequently found in the stomach, when the small bowel is involved, it can lead to various forms of invagination, including complicated or recurrent forms. This case highlights an uncommon presentation of an IFP causing ileo-ileal intussusception in an adult patient, emphasizing the importance of maintaining a high index of suspicion for rare benign causes of bowel obstruction. Although preoperative imaging, especially computed tomography, is crucial for suggesting intussusception, definitive diagnosis of IFP is made histopathologically. The cornerstone for treatment is surgical resection, which has been reported laparoscopically in several studies. Despite their benignity, the increasing number of reported cases further sharpens our understanding of their clinical behavior and management strategies. This requires ongoing documentation and study of these tumors to further enhance early recognition and optimize outcomes of affected patients.
